# Pylephlebitis With Splenic and Mesenteric Vein Thrombosis in a Patient With Diverticulitis

**DOI:** 10.7759/cureus.28524

**Published:** 2022-08-29

**Authors:** Syed Alishan Nasir, Ethan Chambers, Steven Wojkiewicz

**Affiliations:** 1 Internal Medicine, Norwalk Hospital, Norwalk, USA; 2 Radiology, Norwalk Hospital, Norwalk, USA

**Keywords:** therapeutic anticoagulation, mesenteric and portal veins thrombosis, abdominal sepsis, portal vein pylephlebitis, sigmoid diverticulitis

## Abstract

Diverticulitis is a common gastrointestinal complaint that refers to inflammation of colonic diverticula. Its incidence has increased partly due to the increase in prevalence of diverticulosis, which results from poor dietary habits and chronic constipation. An acute diverticulitis episode can vary in severity, ranging from outpatient management of mild abdominal discomfort to inpatient admission requiring emergent surgery. Some common complications associated with diverticulitis include bowel wall perforation, microperforation, abscess formation, bowel obstruction, and colonic fistulas. A lesser-known complication of diverticulitis is pylephlebitis. Pylephlebitis refers to thrombosis of the portal vein resulting from sepsis secondary to an intra-abdominal or pelvic infection. Initially thought to be most associated with appendicitis, literature has emerged that implicates diverticulitis as the most likely culprit. Less frequently, pylephlebitis can also include thrombosis of the abdominal vasculature that drains into the portal vein such as the mesenteric veins and splenic vein. Despite antibiotic therapy, mortality in patients with pylephlebitis is high as it can lead to bowel ischemia, liver failure, or liver abscesses. While antibiotic therapy is the mainstay of treatment, anticoagulation can also be used in conjunction, especially when thrombosis extends beyond the portal vein. Herein, we present a case of a patient who was diagnosed with pylephlebitis with thrombosis extension into the splenic and mesenteric veins, which resulted from an episode of severe sigmoid diverticulitis. Our patient was treated medically with antibiotics and anticoagulation and underwent a loop transverse colostomy with full recovery. He was discharged with intravenous antibiotics and long-term anticoagulation. We present this case to highlight a rare complication of an otherwise common pathology and describe our management that led to a positive outcome for this patient.

## Introduction

Pylephlebitis or suppurative thrombosis of the portal mesenteric venous system is a rare and potentially lethal complication of an intra-abdominal inflammatory process such as diverticulitis, appendicitis, pancreatitis, and inflammatory bowel disease [1-3]. With an estimated mortality rate of greater than 20%, pylephlebitis has been reported to complicate less than 0.2% of all intra-abdominal infections and is reported to have an estimated annual incidence of 0.37-2.7 cases per 100,000 inhabitants per year [3]. Pylephlebitis begins with thrombophlebitis of small veins draining an area of infection. Extension of the thrombophlebitis into larger veins leads to septic thrombophlebitis of the portal vein, which can extend further to involve the mesenteric veins [2-4]. Clinical symptoms are nonspecific, but fever and abdominal pain remain as the two most common symptoms and are present in majority of patients. Patients can also present with complaints related to the abdominal/pelvic process. Diagnosis is made with imaging studies primarily, a computer tomography (CT) scan, or magnetic resonance imaging (MRI). Treatment is established mainly with intravenous (IV) antibiotics; however, anticoagulation use has been reported frequently in the literature.

## Case presentation

**Figure 1:**
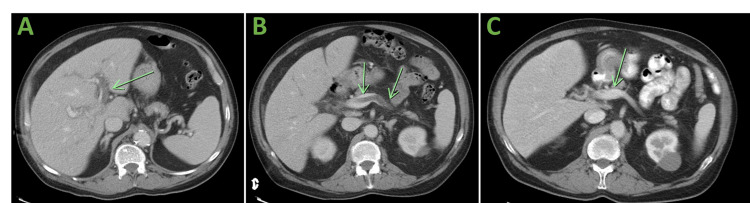
Axial sections showing thrombosis in the portal vein and splenic vein with resolution. (A) Left portal vein thrombosis. (B) Main portal vein and Splenic vein thrombosis. (C) Resolution of portal vein thrombosis after anticoagulation and antibiotic treatment.

A 72-year-old male with a past medical history of gastritis presented to the hospital with fever, abdominal pain, distention, constipation, and hiccups. On admission, he was noted to be afebrile and hemodynamically stable. His laboratory studies were remarkable for a neutrophilic predominant leukocytosis of 29.6 x 10^9^/L, lactic acidosis of 3.5 mmol/L, a predominantly cholestatic transaminitis with an alkaline phosphatase (ALP) of 776 IU/L, gamma-glutamyl transferase (GGT) of 610 U/L, and a total bilirubin of 3.9 mg/dL. A computed tomography (CT) scan showed extensive colonic diverticula with significant inflammation of the sigmoid colon and extensive thrombi of the inferior mesenteric vein, splenic vein, portal vein, and their respective tributaries. The portal vein thrombosis is shown in Figure 1.

The patient was started on ceftriaxone and metronidazole, and due to concern for underlying hypercoagulability, he received a loading dose of IV heparin for anticoagulation. Initial blood cultures obtained showed growth of *Eikenella corrodens*. Infectious disease service was consulted, and the patient’s antibiotic regimen was switched to piperacillin-tazobactam. Due to the diagnostic uncertainty, vascular surgery service was also consulted, which recommended continuing heparin infusion along with performing a hypercoagulable workup. Throughout his hospital stay, our patient’s symptoms continued to worsen, prompting a repeat CT scan, which showed worsening occlusion of the left portal vein and its branches, which were previously only partially occluded. No change was seen in the thrombosis of the other vessels. After completing four days of treatment with piperacillin-tazobactam, our patient developed evidence of drug-induced liver injury due to which his antibiotic coverage was changed to meropenem.

**Figure 2:**
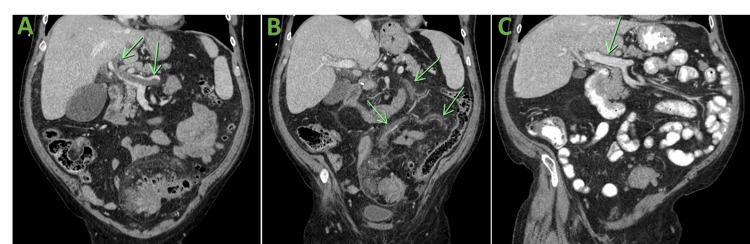
Coronal sections showing thrombosis in the main portal vein, splenic vein, and mesenteric veins with resolution. (A) Thrombosis in the main portal vein and splenic vein. (B) Thrombosis in the inferior mesenteric vein and branches. (C) Resolution of thrombosis in the main portal vein and splenic vein.

At around day 16 of hospitalization, a repeat CT scan was obtained, which showed no change in thrombus in the left portal vein, main portal vein, and proximal splenic veins. A new finding noted was a complete occlusion of the inferior mesenteric vein (Figure 2). The CT scan also showed increased free air superior and lateral to the inflamed sigmoid colon that extended along the proximal inferior mesenteric artery. At this time, colorectal surgery service was consulted, and the patient was taken for sigmoid colectomy. Intraoperatively, our patient was noted to have a firm, indurated sigmoid colon with significantly inflamed surrounding mesentery. Due to the friability of tissue and risk associated with surgical removal of the sigmoid colon, the decision was made to postpone the sigmoid resection and perform a loop transverse colostomy to the left upper quadrant. Following the surgical procedure, he was placed back on a heparin drip.

During hospitalization, our patient was tested for a factor V Leiden mutation, a prothrombin gene mutation, and antiphospholipid antibodies, all of which were negative. Further hypercoagulable workup was deferred to an outpatient setting. Following surgery, our patient continued to have improvement in symptoms. His inflammatory markers downtrended, and he was able to tolerate a diet. He was discharged on a six-week course of meropenem therapy and a six-month course of apixaban with outpatient hematology/oncology follow-up.

While in the outpatient setting, no further hypercoagulable workup was performed due to low suspicion for hypercoagulability given the negative history of previous thrombi and the absence of evidence for malignancy. Our patient was therefore diagnosed with pylephlebitis in the setting of severe sigmoid diverticulitis. A repeat CT scan a few weeks following discharge (Figures 1, 2), showed resolving thrombi in the abdominal vasculature. Our patient continues to do well currently with no evidence of bleeding. He will be discontinuing anticoagulation after completing six months of therapy. He completed his antibiotic course and has not had any recurrence of his abdominal symptoms.

## Discussion

Pylephlebitis remains a common but infrequently encountered complication of diverticulitis and poses a high mortality risk. It was first described in 1846 by Waller, and while definitive diagnosis requires percutaneous drainage of purulent material from the portal tree, in practice, the diagnosis is based on a presence of an infectious process with concomitant thrombosis in the portal venous system [4-5]. Epidemiologically, pylephlebitis is a rare complication of intra-abdominal infections, with an estimated annual incidence of 0.37-2.7 cases per 100,000 inhabitants per year [3]. In a 2013 retrospective study by Belhassen-García et al., a total of 7,796 patients with intra-abdominal infections were evaluated from January 1999 to December 2008. Among these, only 0.16% (approximately 13) patients met the criteria for diagnosis of pylephlebitis. Diagnosis of pylephlebitis was established if patients were noted to have evidence of systemic inflammatory response in the setting of an abdominal infection/inflammation and radiological findings suggestive of thrombosis of the portal vein or one of its branches or if percutaneous drainage of purulent material was expressed from the portal vein or one of its branches [5]. Researchers from the same study also concluded that diverticulitis was the most frequent underlying process followed by biliary tract infections and then appendicitis [5].

The pathophysiology is not clearly understood but is attributed to infectious content and inflammatory mediators draining to the portal circulation [2-3]. Infections are usually polymicrobial, but the most common bacteria that are implicated include *Bacteroides fragilis*, *Escherichia coli*, *Proteus mirabilis*, *Clostridium species*, *Klebsiella*, *Pneumococcus*, *Aerobacter*, and *Streptococcus* [6]. Once a diagnosis is established, the mainstay of treatment is IV antibiotics. Empiric coverage with broad-spectrum antibiotics is encouraged, and there are no clear guidelines with regard to the choice of antibiotics. There remains consensus on the fact that the antibiotics should cover enteric facultative Gram-negative bacilli, anaerobes, and aerobic and microaerophilic streptococci. Additionally, blood cultures should be obtained as these have been shown to be positive in up to 61.5% of cases of pylephlebitis [5]. Once blood cultures return, antibiotic coverage can be narrowed to cover the organism specifically. Four to six weeks of antibiotic therapy is generally sufficient, and both IV and oral formulations have been used [6-7].

The role of anticoagulation remains controversial as no randomized controlled studies concerning pylephlebitis have yet been performed [6]. The aim of anticoagulation is to prevent propagation of the thrombosis, attain portal vein patency, and prevent further complications such as portal hypertension. In our patient, we chose to initiate anticoagulation due to the thrombus burden and due to the concern for underlying hypercoagulability and bowel ischemia. In our literature review, we found multiple studies that employed the use of anticoagulation with favorable outcomes [1,2,3,6,7]. Alternatively, we also found studies that did not employ anticoagulation such as a case report published by Lee and Ryu [8]. Kanellopoulou et al.’s study reported that patients with pylephlebitis who received anticoagulation had a favorable outcome compared to those who received antibiotics alone, citing a complete recanalization of 25.7% vs 14.8% (p < 0.05), no recanalization in 5.7% vs 22.2% (p < 0.05), and death in 5.7% vs 22.2% (p < 0.01) [9]. In another study by Naymagon et al., authors reported that anticoagulated patients had significantly higher rates of portal vein thrombosis resolution than non-anticoagulated patients (58% vs. 21%, p = 0.0201). This translated to lower rates of future chronic portal hypertensive symptoms among anticoagulated patients (11% vs. 47%, p = 0.0034) [10]. Similarly, Baril et al reported better outcomes in patients who did receive anticoagulation vs those who did not mainly when comparing patients with intrinsic hypercoagulable states or extensive thrombosis [11]. On the other hand, Plemmons et al. noted a 100% survival rate among patients who received anticoagulation compared to 60% survival among patients who did not receive anticoagulation although this result was not statistically significant [12]. Conversely, a retrospective review performed by Choudhry et al. showed similar mortality rates when antibiotics were used with or without anticoagulation [13]. Similarly, in a retrospective study of 44 cases by Chang et al., the authors reported that in patients with isolated thrombosis in the portal vein and normal clotting function, anticoagulation may be unnecessary [14]. The role of thrombolytics in the treatment of pylephlebitis is also not well-known. In a case report by Sherigar et al. it was concluded that the indication for thrombolytic therapy was similar to that of using anticoagulation, that is, it can be used to prevent the progression of the thrombus and be used if a patient shows signs of portal hypertension, which is a complication associated with higher mortality [15].

At this time, there are no clinical trials that have commented on anticoagulation duration or have compared the efficacy of different types of anticoagulation regimens in patients with pylephlebitis [16]. Historically, patients have been treated with heparin, low molecular weight heparin, warfarin, and novel oral anticoagulants (NOACs), with a recent increase in the use of NOACs [16-17].

## Conclusions

Pylephlebitis poses the risk of high mortality in patients with diverticulitis. Fortunately, patients who are managed appropriately tend to have good overall prognosis. Clinicians treating diverticulitis in inpatient settings should be weary of pylephlebitis as a potential complication, especially if there is new onset or concomitant transaminitis, worsening abdominal pain, or evidence of ischemic colitis. Interestingly, patients who are diagnosed with pylephlebitis tend to have positive bacterial blood cultures, which suggests that this may serve as a potential risk factor in itself. Empiric broad-spectrum antibiotics are sufficient for initial treatment with subsequent narrowing based on culture data and establishing source control for infection. Based on our literature review and patient course, we recommend evaluating the need for anticoagulation on a case-by-case basis. Factors that would favor the use of anticoagulation include extent of thrombosis, hypercoagulability either due to underlying malignancy or clotting factor disorder, evidence of portal hypertension, or evidence of bowel ischemia. While the literature does not show consensus with regard to the type of anticoagulation to use, we chose to use IV heparin as our anticoagulant mainly due to the short half-life and ability to discontinue easily. This proved to be an important decision as when the need for surgical intervention arose, our patient was easily taken off of his heparin infusion and was prepped for surgery. This serves as another important aspect of this case since severe diverticulitis can lead to complications warranting emergent surgery, and as such, having a patient on an easily reversible anticoagulant can prove to be beneficial and can prevent unnecessary delays in surgical intervention.
